# Hair Follicles as Micro-Organs: MicroRNA-Mediated Control of Growth, Cycling, and Fiber Traits

**DOI:** 10.3390/biom16040504

**Published:** 2026-03-27

**Authors:** Mengsi Xu, Rongyin Zhang, Gao Gong, Shangquan Gan, Wenxin Zheng

**Affiliations:** 1 College of Animal Science, Xinjiang Agricultural University, Urumqi 830052, China; xumengsi100@163.com (M.X.); ggao1995@xjau.edu.cn (G.G.); 2 Institute of Animal Husbandry and Veterinary Medicine, Xinjiang Academy of Agricultural and Reclamation Science, Shihezi 832000, China; 3 College of Animal Science and Technology, Shihezi University, Shihezi 832000, China; 4 Xinjiang Uyghur Autonomous Region Academy of Animal Sciences, Urumqi 830011, China; huaer901@163.com; 5 College of Coastal Agricultural Sciences, Guangdong Ocean University, Zhanjiang 524088, China

**Keywords:** miRNAs, sheep, goat, hair follicle development, regulatory role

## Abstract

Hair follicles are highly specialized mini-organs within the skin that drive the production of wool and cashmere, traits of major biological and economic importance in sheep and goats. Despite their microscopic size, hair follicles exhibit extraordinary regulatory complexity, integrating genetic programs with seasonal, endocrine, environmental, and epigenetic cues. Although transcriptional networks and signaling pathways underlying follicle morphogenesis and cycling have been extensively investigated, the post-transcriptional mechanisms that fine-tune these processes remain insufficiently understood. MicroRNAs (miRNAs) have emerged as pivotal post-transcriptional regulators that coordinate cell fate determination, lineage commitment, and tissue homeostasis. Growing evidence indicates that miRNAs play essential roles in hair follicle stem cell maintenance, proliferation, differentiation, apoptosis, and organ-level development, functioning through interconnected regulatory networks rather than isolated linear pathways. By modulating the expression of key follicle-determining genes and signaling components, miRNA-mediated regulation shapes follicle formation, cyclic regeneration, and fiber traits. In this review, we synthesize recent advances in miRNA research related to hair follicle biology, with a particular focus on wool- and cashmere-bearing mammals. We integrate findings across species to propose a systems-level framework in which miRNA networks interface with canonical signaling pathways and epigenetic mechanisms to orchestrate follicle development and regeneration. Conserved and species-specific regulatory principles are discussed to bridge fundamental follicle biology with practical applications in fiber production. Overall, this review highlights miRNAs as a critical yet previously underappreciated regulatory layer in hair follicle biology. A deeper understanding of miRNA-mediated control provides new conceptual insights into wool and cashmere development and offers a foundation for future molecular breeding and precision regulation strategies in livestock.

## 1. Introduction

Sheep and goats were among the earliest domesticated livestock species and continue to play essential roles in global agriculture [1]. In addition to meat and milk production, fiber-producing ruminants—including wool sheep and cashmere goats—contribute substantially to the textile industry through the production of natural fibers, such as wool and cashmere [2]. Fiber quality is determined by multiple phenotypic traits, including diameter, curvature, length, and pigmentation. As global demand for high-quality natural fibers increases, breeding strategies have increasingly focused on improving both fiber yield and fineness [3,4].

Cashmere-producing goats, particularly in countries such as China, Mongolia, and Iran, represent an economically important subgroup within fiber-producing livestock. In recent decades, advances in breeding and molecular research have aimed to elucidate the biological mechanisms underlying fiber growth and trait variation [5].

Mammalian skin, composed primarily of the epidermis and dermis, serves as the outer protective barrier of the body [6]. In fiber-producing species such as sheep and goats, hair (wool or cashmere) represents a key economic trait. Hair fibers originate from hair follicles, which are specialized epidermal appendages whose structural and functional characteristics directly determine fiber growth, yield, and quality [7]. Hair follicle morphogenesis and cycling are tightly regulated biological processes involving coordinated epithelial–mesenchymal interactions and dynamic signaling activity [8].

Hair growth is influenced by environmental conditions, nutritional status, metabolic factors, and endocrine signals; however, genetic and molecular regulatory mechanisms play a central role in determining follicle development and fiber traits. Understanding the molecular basis of hair follicle regulation is therefore essential for improving fiber quality and production efficiency through modern breeding strategies [9].

## 2. Structure and Developmental Features of Animal Skin and Hair Follicles

### 2.1. Anatomy and Cellular Composition of Animal Skin and Hair Follicles

The skin, the largest organ of the body, provides a barrier against environmental insults while preventing transepidermal fluid loss. Histologically, it is composed of three major layers: the epidermis, dermis, and subcutaneous tissue. The epidermis consists of stratified keratinized epithelium organized into distinct layers: stratum corneum, lucidum, granulosum, spinosum, and basale. Beneath the epidermis, the collagen-rich dermis contains vasculature, nerve fibers, and sensory receptors. The subcutaneous layer is composed predominantly of adipose tissue [10]. Within the dermis, two functionally distinct regions can be identified: the papillary (hair follicle-associated) layer and the reticular layer. The former harbors hair follicles and associated appendages, including sebaceous and sweat glands, which collectively contribute to thermoregulation, ultraviolet protection, microbial defense, mechanosensation, and social signaling [11,12].

**Figure 1:**
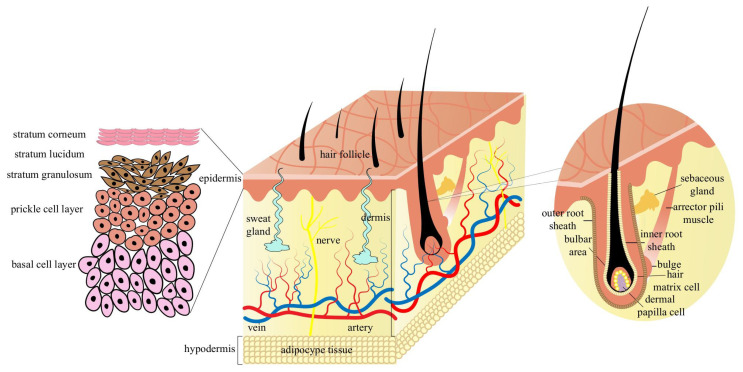
Architecture of the mammalian skin and hair follicles.

Mammalian hair, comprising the hair shaft, root and bulb, is a defining integumentary feature. The hair shaft consists of highly differentiated keratinized cells enriched in keratin and keratin-associated proteins [13]. Hair is generated by hair follicles, miniaturized, self-renewing skin appendages formed by epithelial–mesenchymal interactions at the follicular base [14]. Structurally, the follicle is organized concentrically into the outer root sheath (ORS), companion layer, inner root sheath (IRS) and hair shaft [15] (Figure 1). Hair follicles contain more than twenty cell types, broadly categorized as epithelial or dermal in origin [7,16]. Epidermal epithelial components include hair follicle stem cells (HFSCs), ORS cells, and matrix (Mx) cells. Matrix cells proliferate and differentiate to generate IRS cells and the medulla, cortex, and cuticle of the hair shaft [17,18]. Dermal components, including dermal papilla cells (DPCs) and dermal sheath cells, function as specialized mesenchymal regulators. Although they do not directly form hair fibers, they secrete molecular cues that regulate epithelial proliferation, differentiation, and apoptosis [19,20,21]. Notably, DPC and dermal sheath cells exhibit functional plasticity and may interconvert during distinct stages of follicle development, thereby contributing to regenerative capacity [22].

HFSCs, localized primarily within the bulge region, serve as multipotent progenitors for follicular lineages [23]. At the base of the follicle resides the hair bulb, where DPCs form a signaling niche surrounded by rapidly proliferating matrix cells. Molecular signals derived from DPCs regulate matrix–cell proliferation and differentiation, thereby orchestrating hair shaft and sheath formation [24,25]. Coordinated epithelial–mesenchymal crosstalk thus governs follicle morphogenesis, cyclic remodeling, and ultimately fiber traits such as diameter and pigmentation.

### 2.2. Morphogenetic Events in Hair Follicle Formation

**Figure 2:**
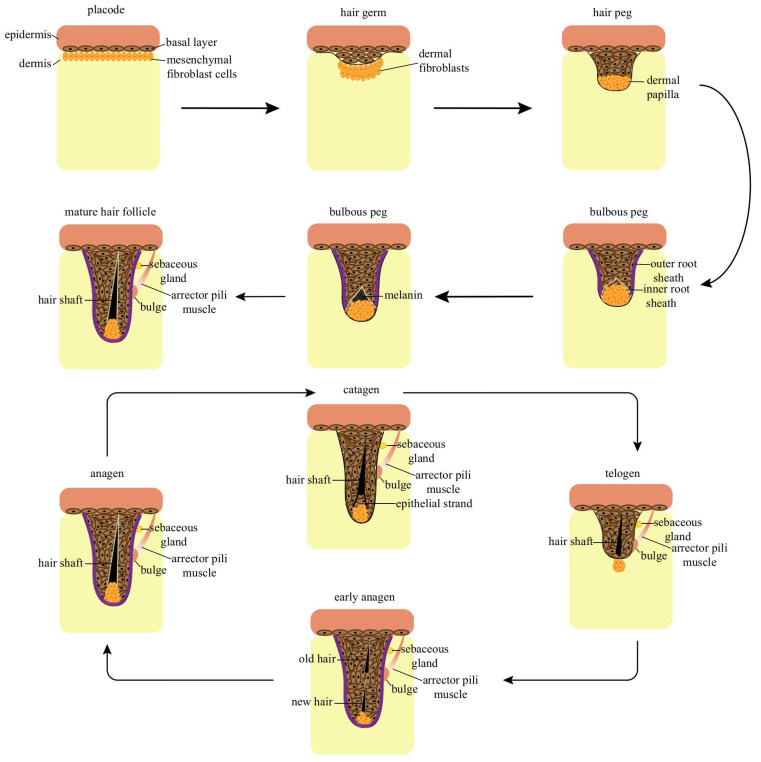
Morphogenetic foundations and cyclic renewal of hair follicles.

Hair follicle development is initiated during embryogenesis and is largely completed before or shortly after birth. Thereafter, follicles undergo cyclical regeneration, driving repetitive phases of growth, regression, and rest [13,26] (Figure 2).

In fiber-producing ruminants, hair follicles are classified as primary or secondary. Primary follicles arise from coordinated epithelial–mesenchymal interactions during early development, whereas secondary follicles emerge subsequently and are responsible for fine fiber production. In cashmere goats, secondary follicles generate cashmere fibers, whereas primary follicles produce coarse guard hairs [27]. The secondary-to-primary follicle ratio (S/P ratio) serves as an important quantitative determinant of fiber fineness and yield.

From a morphological perspective, follicle morphogenesis proceeds through eight defined stages driven by reciprocal epithelial–dermal signaling [28,29]. Epidermal keratinocytes initially thicken to form a placode, while underlying mesenchymal cells condense to establish the dermal papilla. This reciprocal signaling promotes downgrowth of the epidermis and formation of the hair germ. The hair germ subsequently elongates into a hair peg, and keratinocytes progressively surround the dermal papilla. During later stages, differentiation generates concentric layers—including the IRS and hair shaft components (medulla, cortex, and cuticle)—while the ORS forms a protective outer structure [28,30,31].

### 2.3. Cyclic Regeneration: The Hair Follicle Growth Cycle

Mature hair follicles undergo lifelong cycles consisting of anagen (growth), catagen (regression), and telogen (rest) [7]. These transitions involve extensive structural remodeling and dynamic shifts in cellular activity, regulated by signaling pathways, transcription factors, and epigenetic mechanisms [32]. After completing their initial development, mature hair follicles enter a continuous cycle of regeneration. This cycle is divided into three phases based on structure and growth activity: anagen (hair growth is rapid), catagen (gradual cessation of hair growth), and telogen (complete cessation of hair growth) [7].

The structure of the hair follicle is most complete during anagen. HFSCs located in the bulge region are activated and begin to proliferate. They then migrate downward along the ORS, eventually forming the hair Mx cells that encapsulate the DP area. Upon receiving molecular signals from DPCs, hair Mx cells rapidly proliferate, differentiate, and migrate upward, progressively forming the six cell lineages of the IRS and the hair shaft. As the cells comprising the hair shaft differentiate, they expel their organelles and form a rigid structure through the physical cross-linking of cystine-rich keratins into intermediate filaments. Concurrently, the IRS keratinizes, forming a rigid conduit that guides the hair shaft toward the skin surface. Mx cells undergo a limited number of divisions before differentiating. Once their proliferative capacity is exhausted, differentiation slows, and the follicle enters the catagen period [33,34,35,36,37].

Catagen is a transition marked by programmed regression of the lower, cycling portion of the follicle. Lacking cellular renewal, this segment atrophies. HFSCs cease differentiation, growth stops, and the follicle shortens as the hair bulb retracts upward into the permanent region [38,39,40]. Telogen is the final, resting stage of the cycle. The cycling portion has fully regressed, and cellular proliferation within the follicle ceases. HFSCs remain quiescent, awaiting activation signals. The old hair shaft, now a club hair, loosens and is eventually shed. As telogen ends, the bulge region, where HFSCs reside, comes into close proximity with the DPCs. DPCs then release signals that activate the stem cells, prompting proliferation and initiating the next anagen phase [41,42].

## 3. miRNA Biology: Fundamental Concepts and Relevance to Hair Follicles

Although small, the hair follicle is a structurally and functionally complex mini-organ whose morphogenesis depends on coordinated interactions among stem cells, cell-cycle regulators, and multiple signaling pathways [43]. Follicle formation is not governed by a single dominant gene but instead emerges from layered, quantitative control across gene networks. Such distributed regulation complicates direct genetic manipulation and underscores the need to understand higher-order regulatory mechanisms that integrate polygenic inputs [44].

MicroRNAs (miRNAs) are particularly well suited to this role. By engaging one-to-many and many-to-one targeting architectures, miRNAs modulate gene networks rather than isolated transcripts—an organizational logic that parallels the polygenic control of follicle morphogenesis [45]. Their regulatory architecture enables graded control of gene expression amplitude and timing, features central to cyclic follicle growth. Since the first miRNA was identified in *Caenorhabditis elegans* in 1993 [46], followed by the discovery of let-7 in 2000 [47], the field has expanded rapidly. Thousands of miRNAs have since been cataloged across species, accompanied by progressive refinement of their mechanistic understanding. Early models emphasized miRNA binding to the 3′ untranslated region (3′UTR) of target mRNAs, leading to transcript degradation or translational repression. Subsequent work demonstrated that miRNA binding sites may also reside within coding sequences (CDS), 5′UTRs, and even promoter regions, broadening their regulatory scope. In addition to mRNA destabilization, miRNAs may inhibit translation, modulate transcription factor networks indirectly, and participate in intercellular communication via extracellular vesicles [48]. These properties—network-level modulation, temporal plasticity, and capacity for fine quantitative control—make miRNAs central to hair follicle morphogenesis, cycling, and lineage specification.

### 3.1. Overview and Characteristics of miRNAs

miRNAs are short non-coding RNAs (ncRNAs) that post-transcriptionally regulate gene expression. They are widely distributed throughout eukaryotic genomes, frequently located within introns, intergenic regions, or occasionally with 3′UTRs of coding genes. The 5′ seed region (nucleotides 2–8) is critical for target recognition, whereas additional pairing outside the seed can stabilize binding but is often dispensable [49]. miRNAs exhibit strong evolutionary conservation while maintaining spatiotemporal specificity. Their expression varies across tissues and developmental stages [50,51]. Importantly, miRNA–mRNA interactions are rarely one-to-one. A single miRNA can regulate hundreds of transcripts, and conversely, one mRNA can be targeted by multiple miRNAs [52,53]. Genome-wide analyses estimate that 60–70% of protein-coding genes are subject to miRNA regulation [54]. Through this network architecture, miRNAs shape proliferation, apoptosis, differentiation, and developmental transitions [55].

### 3.2. Biological Biogenesis: From Nuclear Transcription to Cytoplasmic Maturation

**Figure 3:**
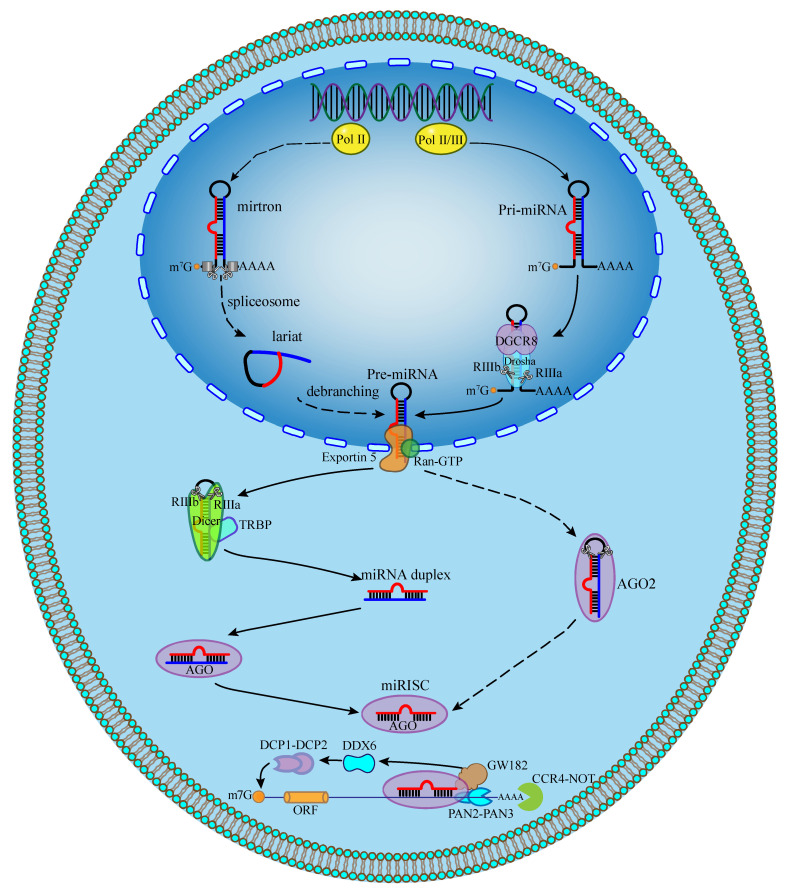
Biogenesis and functional mechanism of miRNAs: nuclear processing, cytoplasmic maturation, and target regulation. pri-miRNA: primary miRNA transcript; RIII: ribonuclease III; RISC: RNA-induced silencing complex; AGO: Argonaute; DGCR8: microprocessor complex subunit; DDX6: DEAD-box helicase 6; RNA-GTP: Ran GTPase activating protein 1; m7G: 7-methylguanosine; Pol: RNA polymerase; DCP: Decapping protein; PAN: Poly (A) nuclease; GW182: Glycine-tryptophan protein of 182 kDa; CCR4-NOT: Carbon catabolite repression 4—negative on TATA-less; TRBP: TAR RNA-binding protein.

Canonical miRNA biogenesis initiates in the nucleus, where most miRNA genes are transcribed by RNA polymerase II as capped and polyadenylated pri-miRNAs containing stem-loop structures [56]. These transcripts are processed by the microprocessor complex composed of the RNase III enzyme Drosha (~160 kDa) and its RNA-binding cofactor DGCR8, which together excise ~70 nt pre-miRNAs from the pri-miRNA hairpin [57,58,59]. DGCR8 mediates substrate recognition and nuclear localization, whereas the tandem RNase III domains (RIIIa/RIIIb) of Drosha execute staggered cleavage. Subsequently, pre-miRNAs are exported to the cytoplasm via Exportin-5 in a Ran-GTP-dependent manner; GTP hydrolysis promotes cargo release [60,61]. In the cytoplasm, Dicer (~281 kDa) recognizes the 3′ overhang through its PAZ domain and cleaves the stem-loop to generate ~22-nt miRNA duplexes [62,63]. One strand (−5p or −3p) is preferentially loaded onto Argonaute to form the AGO-RISC complex, which associates with endoplasmic reticulum-bound ribosomes and accessory factors such as AMP1 to repress translation or facilitate mRNA decay [64] (Figure 3).

In addition to this canonical pathway, alternative routes diversify miRNA maturation. Mirtrons, generated from spliced introns, bypass Drosha cleavage and enter the pathway at the pre-miRNA stage before Dicer processing [65,66]. Conversely, miR-451 undergoes Dicer-independent maturation through direct AGO2-mediated cleavage followed by exonucleolytic trimming [67,68]. Genetic ablation and deep-sequencing analyses further demonstrate partial redundancy among Drosha, Dicer, and Exportin-5: although Drosha loss markedly reduces canonical miRNAs, subsets persist in Dicer-deficient cells, and Exportin-5 depletion exerts limited global effects, supporting the existence of compensatory processing or export mechanisms [69]. Importantly, these mechanistic insights are primarily derived from biochemical assays, targeted gene disruption in cell lines, and sequencing-based profiling, whereas conditional or tissue-specific in vivo models remain comparatively limited. Collectively, current evidence supports a modular and context-dependent network of miRNA biogenesis rather than a strictly linear cascade.

### 3.3. Mechanisms of Gene Silencing: mRNA Decay and Translational Repression

The primary mechanisms by which miRNAs regulate gene expression are through binding to target mRNAs and inducing translational repression or mRNA decay. This interaction is most commonly mediated by the miRNA seed sequence binding to complementary sites in the 3′UTR of the target mRNA [70,71]. However, functional miRNA binding sites have also been identified in the 5′UTR and the coding sequence (CDS) of some genes [72,73], where they can, in rare cases, even upregulate protein expression. Additionally, miRNAs can be packaged into exosomes and secreted, mediating intercellular communication.

The silencing of target mRNAs occurs mainly through two downstream processes: mRNA decay and translation inhibition. AGO proteins, bound to the miRNA, recruit members of the GW182 protein family. GW182 proteins interact with poly (A)-binding protein (PABPC) and recruit deadenylase complexes, primarily the CCR4-NOT complex and, to a lesser extent, PAN2-PAN3. Deadenylation of the mRNA tail is a signal for the removal of the 5′ 7-methylguanosine cap by the DCP1-DCP2 decapping complex, which is also recruited by GW182 and associated factors like DDX6. Once decapped, the mRNA is rapidly degraded by the 5′-to-3′ exoribonuclease 1 (XRN1) [74,75,76,77].

## 4. miRNA-Mediated Regulation of Hair Follicle Development in Mouse Models

### 4.1. miRNAs in Mouse Hair Follicle Cells: Proliferation, Differentiation, and Stem Cell Dynamics

Mouse hair follicle morphogenesis and cycling represent a dynamic epithelial–mesenchymal system governed by stem cell plasticity and tightly coordinated signaling networks. Owing to the availability of genetic models, this system has become a powerful in vivo platform for dissecting post-transcriptional regulatory mechanisms, including those mediated by miRNAs. Pickup et al. reported that miR-148a regulates hair follicle activity by modulating *ROCK1* and *ELF5* expression [78]. Their conclusions were supported by target prediction and molecular assays assessing gene expression following miR-148a inhibition. Suppression of miR-148a increased *ROCK1* and *ELF5* levels and was associated with enhanced epidermal stem cell proliferation and hair lineage commitment. Notably, the mechanistic inference relies primarily on gene expression and functional manipulation rather than conditional in vivo genetic deletion.

miR-214 displays dynamic spatial and temporal expression in mouse skin epithelium [79]. In transgenic mice overexpressing miR-214, hair follicle cell proliferation was reduced, follicle numbers decreased, and hair bulb size diminished. Complementary in vitro experiments in primary mouse epidermal keratinocytes (PMEKs) demonstrated suppressed proliferation upon miR-214 overexpression. These findings are supported by both in vivo transgenic models and in vitro gain-of-function assays. Nagosa et al. combined functional assays with direct target validation to investigate miR-184 [80]. Dual-luciferase reporter assays confirmed that *K15* and *FIH1* are direct targets of miR-184. Functional analyses showed that miR-184 suppresses epidermal stem cell self-renewal while promoting differentiation, thereby providing mechanistic evidence beyond correlative expression data. Ge et al. characterized the spatiotemporal expression of the miR-29a/b1 cluster in mouse HFSCs during distinct hair cycle stages [81]. Using transgenic overexpression and inhibition models, they demonstrated that miR-29a/b1 impairs HFSC proliferation and differentiation. Bmpr1a, a BMP pathway component, was identified as a functional target, linking miR-29a/b1 to modulation of BMP signaling and follicular homeostasis. These conclusions are supported by in vivo genetic models together with pathway-associated molecular analyses.

Genetic loss-of-function evidence underscores the essential role of miR-205 in skin development. miR-205 deficiency resulted in perinatal lethality and severe defects in epidermal and follicular morphogenesis, accompanied by impaired maintenance of skin stem cell properties [82]. In contrast, transgenic overexpression of miR-203 in the basal epidermal layer led to perinatal lethality and premature keratinocyte differentiation, indicating that miR-203 dosage critically regulates the proliferation–differentiation balance [83]. Similarly, transgenic mice overexpressing miR-24 exhibited altered follicular architecture and reduced hair density [84]. Reduced *Tcf-3* expression was observed in these models, suggesting that miR-24 influences Wnt-associated transcriptional regulation; however, pathway activity was inferred primarily from downstream expression changes. Downregulation of miR-30a-5p was reported to suppress HFSC apoptosis and promote proliferation, accompanied by increased β-catenin expression [85]. In this case, activation of the Wnt/β-catenin pathway was inferred indirectly from protein expression rather than demonstrated through direct functional pathway assays.

### 4.2. Crosstalk Between miRNAs and Signaling Pathways in Mouse Hair Follicle Cycling

In addition to regulating cellular proliferation and differentiation, miRNAs modulate signaling pathways that orchestrate hair follicle cycling. Importantly, the strength of mechanistic evidence varies across studies. In the case of miR-214, phenotypic alterations observed in transgenic overexpression models [79] were complemented by dual-luciferase reporter assays demonstrating that *β-catenin* is a direct target. These data establish a mechanistic link between miR-214 and Wnt signaling, supported by both in vivo and molecular validation.

miR-205 has been reported to target multiple genes involved in actin cytoskeleton organization [86]. Functional studies indicate effects on cell cycle progression and hair regrowth; however, most supporting evidence derives from gene expression profiling and in vitro assays rather than conditional pathway-specific in vivo models. Ge et al. further demonstrated, using miR-29a/b1 gain- and loss-of-function mouse models, that this miRNA cluster suppresses WNT signaling, BMP signaling, and HFSC cycle progression [81]. These findings were supported by pathway-related gene expression analyses, although direct biochemical pathway activity assays were limited.

miR-218-5p has been shown to promote hair follicle development, with molecular evidence indicating repression of the WNT inhibitor SFRP2 and concomitant elevation of β-catenin levels [87]. While target repression was experimentally supported, activation of WNT signaling was primarily inferred from downstream expression changes. miR-21 has been reported to modulate BMP pathway-associated inhibitory genes, including *PTEN*, *PDCD4*, *TIMP3*, and *TPM1*, thereby influencing proliferation and migration of primary mouse keratinocytes [88]. These conclusions are based largely on in vitro gain- and loss-of-function experiments. Shi et al. provided direct mechanistic evidence that miR-31-5p targets the 3′UTR of RASA1 using luciferase reporter assays [89]. Functional analyses demonstrated increased ERK1/2 activity following miR-31-5p overexpression, linking miRNA-mediated post-transcriptional regulation to activation of the RAS/MAPK pathway in mouse skin.

**Figure 4:**
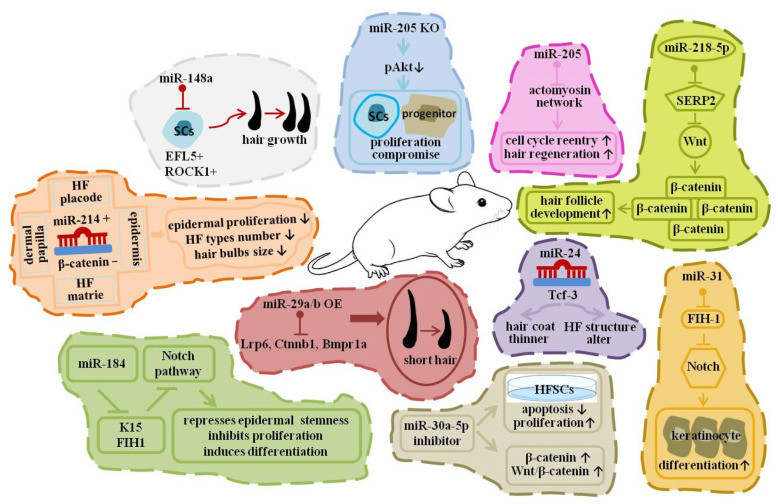
Decoding miRNA-mediated regulation of hair follicle development in mouse models. An upward arrow indicates increased biofunction, and a downward arrow indicates decreased biofunction.

Mardaryev et al. identified *DLX3* and *FGF10* as direct targets of miR-31 through reporter assays and gene expression analyses [90]. Their data indicate that miR-31 modulates components of both Wnt and BMP signaling pathways, contributing to balanced regulation of hair follicle growth and hair shaft formation. Additional studies suggest that miR-31 negatively regulates *HIF-1* expression and may influence Notch pathway activity, thereby promoting keratinocyte differentiation [91]. However, the extent to which these interactions represent direct versus indirect regulatory effects remains to be fully clarified (Figure 4).

Collectively, mouse genetic models—ranging from transgenic overexpression and knockout systems to in vitro functional assays and luciferase-based target validation—demonstrate that miRNAs regulate hair follicle development at multiple levels. Distinguishing among correlative expression patterns, molecular target validation, cellular functional assays, and in vivo genetic models is essential for accurate interpretation of causality. Such stratification of evidence strengthens mechanistic understanding and enhances the translational relevance of these findings for regenerative medicine and hair disorder therapeutics.

## 5. miRNAs in Human Hair Follicle Biology: Implications for Health and Disease

At birth, humans possess approximately five million hair follicles, and folliculogenesis is largely completed during embryogenesis [92]. Unlike certain lower vertebrates, mammals exhibit minimal postnatal follicle neogenesis. Consequently, understanding the molecular mechanisms governing follicle maintenance, cycling, and degeneration is essential for addressing hair disorders. Direct experimental manipulation of human follicles is constrained by ethical and technical limitations. Nonetheless, increasing numbers of transcriptomic and functional studies using human tissues and primary cells have begun to delineate miRNA-mediated regulatory networks relevant to follicular homeostasis and disease.

Mohammadi et al. performed comparative profiling of hair follicle stem cells and progenitor cells from healthy individuals and patients with androgenetic alopecia (AGA) [93]. Differential miRNA signatures were identified between groups. In healthy follicles, specific miRNAs correlated with pathways governing stem cell renewal and differentiation. In AGA samples, aberrant upregulation of certain miRNAs was associated with reduced expression of genes implicated in follicle growth. Importantly, much of this evidence is derived from expression profiling and correlation analyses, and causal regulatory relationships require further functional validation. miR-205 is broadly expressed in the human epidermis and has been reported to modulate Akt activity and suppress apoptosis [94]. These conclusions are based primarily on molecular and cellular assays, rather than conditional in vivo genetic models. Wei et al. showed that miR-203 is detectable in the epidermal basal layer at 17 weeks of gestation, with conserved localization in adult skin [95]. Functional manipulation experiments indicated that premature miR-203 expression reduces basal keratinocyte proliferative potential, whereas its inhibition enhances proliferation. These data are largely derived from in vitro gain- and loss-of-function assays. Deng et al. conducted miRNA microarray analyses in AGA patients and identified multiple differentially expressed miRNAs relative to controls [96]. Among these, miR-133b was further examined in functional assays. Experimental modulation suggested that miR-133b influences genes associated with follicle growth; however, most mechanistic interpretations are based on in vitro experiments and pathway-associated gene expression changes.

Similarly, inhibition of hsa-miR-136-5p in human DPCs enhanced proliferation and altered cell cycle-related protein expression [97]. These findings are derived from cultured DPC models, indicating potential therapeutic relevance but not yet supported by in vivo human genetic evidence.

Beyond cellular proliferation, several studies suggest that miRNAs intersect with canonical signaling pathways implicated in follicle biology. miR-214 has been reported to inhibit the proliferation and differentiation of human hair follicle stem cells by reducing *EZH2* expression and attenuating Wnt/β-catenin signaling [98]. Evidence includes target validation and in vitro functional assays; however, pathway modulation was inferred largely from downstream molecular readouts. miR-148 has been associated with enhanced proliferation of human hair follicle stem cells, with data suggesting involvement of the Wnt pathway [99]. Here again, pathway regulation was inferred from gene expression analyses rather than direct biochemical measurement of signaling activity. Cao et al. provided functional evidence that miR-100 represses multiple Wnt pathway inhibitors, leading to increased nuclear *β-catenin* levels and elevated *C-MYC* and *Cyclin D1* expression [100]. These findings were supported by molecular assays and pathway activity measurements in cultured cells. miR-140-5p has been reported to suppress BMP2 expression, thereby attenuating BMP signaling and promoting proliferation of human DPCs [101]. Evidence includes target prediction combined with in vitro validation assays. In alopecia areata, Erfan et al. identified differential expression of *NEAT1* and miR-101 in patient samples relative to controls [102]. These findings are primarily diagnostic and correlative, highlighting potential biomarker value rather than mechanistic causality.

Collectively, studies in human tissues and primary cells indicate that miRNAs are integral to follicle maintenance and pathology. However, the majority of mechanistic insights are derived from expression analyses and in vitro functional experiments. Conditional in vivo validation in humanized or organoid systems remains limited.

## 6. miRNA Networks in Goat Hair Follicle Development and Cashmere Production

**Figure 5:**
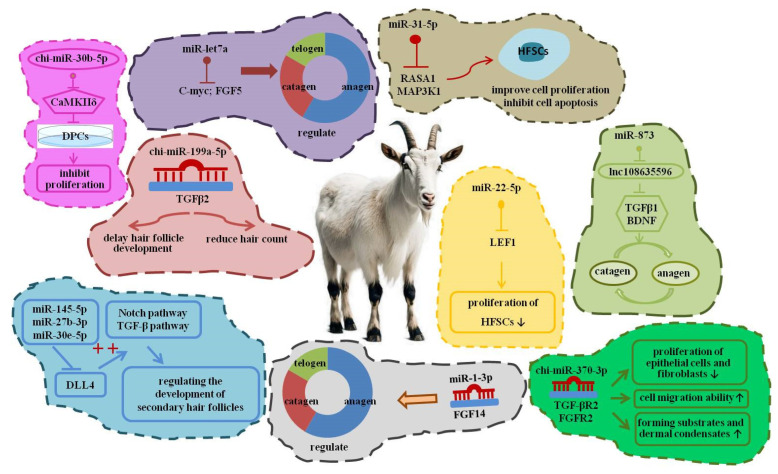
A multilayered miRNA regulatory network orchestrating goat hair follicle morphogenesis, cycling, and fiber traits. An upward arrow indicates increased biofunction, and a downward arrow indicates decreased biofunction.

Hair follicle morphogenesis in goats follows conserved mammalian principles, with embryonic specification followed by cyclical postnatal growth. In cashmere goats, secondary hair follicles are responsible for fine fiber production, whereas primary follicles produce coarse fibers. Increasing evidence indicates that miRNAs contribute to follicle cycling, stem cell regulation, and fiber trait determination (Figure 5).

### 6.1. miRNA–Target Gene Regulatory Networks in Goat Hair Follicle Cycling

Transcriptomic studies demonstrate that miRNAs and their target genes form complex regulatory networks during goat follicle cycling. Ma et al. reported that miR-let-7a exhibits stage-specific expression during anagen and catagen in cashmere goats and regulates *C-MYC* and *FGF5* expression. Evidence includes expression profiling and functional assays [103]. Genetic evidence from *TGFβ2*-deficient mice shows delayed follicle development and reduced follicle numbers, indicating that *TGFβ2* promotes folliculogenesis [104]. Building on this, Han et al. demonstrated that chi-miR-199a-5p suppresses TGF-*β2* expression in cashmere goats, suggesting indirect modulation of follicle development [105]. Zhang et al. showed, using dual-luciferase reporter assays, that *FGF14* is a direct target of miR-1-3p [106]. Expression patterns across follicle cycle stages support a regulatory role. Ma et al. confirmed through luciferase assays that miR-203 directly binds the 3′UTRs of *DDOST* and *NAE1* and reduces their mRNA and protein levels [107]. Phase-specific expression analysis supports a role in resting follicle regulation. Liu et al. performed integrated miRNA–mRNA association analyses and identified miR-195 and its targets (*CHP1*, *SMAD2*, *FZD6*, and *SIAH1*) as candidate regulators of follicle initiation [108]. These conclusions are primarily derived from correlation and bioinformatic prediction. Multiple studies have constructed ceRNA networks linking lncRNAs, circRNAs, and miRNAs in secondary follicle cycling [109,110,111,112,113,114,115,116,117,118,119,120,121]. In several cases, dual-luciferase assays validated direct miRNA binding (e.g., lncRNA MSTRG.1705.1-Chi-miR-1 [115]; circRNA3236-chi-miR-27b-3p [120]). However, many networks remain computationally predicted and require further functional dissection.

### 6.2. Integration of miRNAs with Key Signaling Pathways in Goat Folliculogenesis

Hair follicle cycling—comprising anagen, catagen, and telogen—is orchestrated by complex signaling networks in which miRNAs serve as critical regulatory nodes. Expression profiling across the caprine hair cycle has systematically linked miRNAs to these pathways. Using Solexa sequencing of Shaanbei white cashmere goat skin, Yuan et al. identified phase-specific miRNA signatures whose predicted targets were significantly enriched in pathways governing follicular dynamics, including MAPK, WNT, TGF-β, Shh, NOTCH, and JAK-STAT, establishing a broad regulatory landscape for miRNAs in secondary follicle cycling [122]. Comparative analysis between cashmere goats and fine-wool sheep further revealed 1246 known and 107 novel differentially expressed miRNAs (DE miRNAs), with KEGG enrichment implicating WNT and MAPK as primary pathways targeted by these miRNAs [123]. Spatiotemporal expression patterns provide additional functional insights. Su et al. identified five novel miRNAs in cashmere goat skin, noting that chi-miR-421* and chi-miR-421 peaked during October (follicular proliferation), whereas chi-miR-2284n, chi-miR-1839, and chi-miR-374b were maximally expressed in February (quiescence). Notably, chi-miR-421* and chi-miR-1839 target *CSNK1A1* and *WDR12*, respectively, positioning them as modulators of Wnt/Notch signaling during phase transitions [124]. Developmental transcriptomics of fetal cashmere goat skin (days 45, 55, 65, and 75) by Shang et al. identified 66 miRNAs associated with SF morphogenesis. Predicted interactions—including miR-145-5p-*DLL4*, miR-27b-3p-*DLL4*, miR-30e-5p-*DLL4*, miR-193b-3p-*TGFβ1*, miR-181b-5p-*NOTCH2*, and miR-103-3p-*NOTCH2*—were significantly enriched in Notch and TGF-β pathways, implicating these miRNAs in SF development [125]. In Zhongwei goats, Ding et al. identified 28 DE miRNAs between embryonic days 45 and 108. Their targets were associated with pathways controlling hair curvature and follicle development, including TGF-β/SMAD, PI3K-Akt, JAK-STAT, and MAPK [126]. Similarly, Zhang et al. performed miRNA sequencing across the cashmere goat hair cycle, identifying 29 upregulated and 32 downregulated miRNAs during anagen relative to telogen. Functional enrichment of their targets revealed significant representation in Wnt, ECM-receptor interaction, and VEGF pathways [127].

Collectively, these studies demonstrate that miRNAs are dynamically expressed throughout hair follicle cycling and morphogenesis, exerting their regulatory influence primarily through modulation of evolutionarily conserved signaling pathways—notably WNT, MAPK, TGF-β, and Notch—that govern epithelial–mesenchymal interactions, cell proliferation, and differentiation during follicular development.

### 6.3. Cell-Specific Functions of miRNAs in Goat Hair Follicle Stem Cells and Dermal Papilla Cells

miRNAs exhibit pronounced cell-type specificity within goat follicles, with validated expression in HFSCs, DPCs, Mx cells, ORS cells, and IRS cells [128]. Given that follicle morphogenesis and regeneration depend on sustained HFSC activity, most studies have focused on HFSC-autonomous regulation. Functional stratification, however, reveals heterogeneous levels of validation. In HFSCs, miR-148a-3p overexpression inhibits differentiation based on in vitro perturbation assays [116]. Gain- and loss-of-function experiments indicate that miR-31-5p promotes proliferation and suppresses apoptosis through *RASA1* targeting and *MAP3K1* upregulation [129]. whereas miR-101 enhances proliferation via *DUSP1* repression [130]. In contrast, miR-145-5p inhibits proliferation and promotes apoptosis by targeting *DUSP6* [131]. miR-149-5p exhibits dual functions: reporter assays support direct targeting of *β-catenin* to promote differentiation [132], while independent experiments demonstrate enhanced proliferation and reduced apoptosis via *CMTM3* regulation [133]. Differential profiling between DPCs and HFSCs identified 13 miRNAs, among which miR-1 suppresses proliferation and promotes differentiation through *IGF1R* and *LEF1* targeting, supported by overexpression studies and target validation assays [134]. Similarly, miR-22-5p negatively regulates HFSC proliferation via *LEF1* repression [135]. Notably, most conclusions are derived from correlative expression analyses combined with luciferase assays and in vitro functional manipulation; conditional genetic models in vivo remain lacking.

CeRNA networks further refine HFSC control. circRNA-1967 enhances *LEF1* expression by sponging miR-93-3p, promoting stem cell differentiation [136], whereas circRNA-0100 modulates *KLF5* transcription through miR-153-3p to drive lineage commitment [137]. These mechanisms are supported primarily by reporter assays and in vitro knockdown/overexpression systems.

Reciprocal epithelial–mesenchymal signaling is similarly modulated by miRNAs. Luciferase-based validation indicates that chi-miR-130b-3p directly targets *WNT10A*, influencing the proliferation of epithelial and dermal cells [138]. chi-miR-370-3p suppresses proliferation and enhances migration via *TGF-βR2* and *FGFR2* targeting, facilitating dermal condensate formation [139]. As the follicular signaling hub [25], DPCs integrate these regulatory inputs. Transcriptomic analyses identified 25 core DPC genes (e.g., *HOXC8*, *RSPO1*) and associated ceRNA axes (XR_310320.3-chi-miR-144-5p-*HOXC8*; XR_311077.2-novel_624-*RSPO1*) implicated in stem cell activation [140]. chi-miR-30b-5p, enriched in telogen, inhibits DPC proliferation via *CaMKIIδ* targeting [127]. Functional assays further validated miR-26a-*Smad1* and miR-130a-*Smad5* interactions, revealing opposing effects on DPC proliferation and TGF-β/SMAD signaling [126]. lncRNA-*H19* promotes DPC proliferation through the chi-miR-214-3p/*β-catenin* axis [141], whereas lncRNA-599547 enhances inductive capacity by sponging miR-15b-5p to derepress *WNT10B* [142].

Collectively, these studies establish that miRNAs govern goat hair follicle biology through cell-type-specific regulation of HFSC maintenance, epithelial–mesenchymal crosstalk, and DPC signaling competence, with ceRNA networks adding further regulatory sophistication.

### 6.4. miRNAs as Regulators of Cashmere Fiber Traits: Fineness, Color, and Yield

Comparative profiling across goat breeds, anatomical skin regions, and distinct follicular stages consistently demonstrates that miRNA expression exhibits species-specific, tissue-specific, and spatiotemporal dynamics in caprine skin. During the hair cycle, miR-148a-3p increases progressively from anagen to catagen and telogen in Liaoning cashmere goats [143]. Longitudinal analyses of fetal skin further showed that members of the miR-200 and let-7 families are upregulated concomitantly with follicle morphogenesis, implicating them in early follicular formation [144]. Stage-dependent expression of miR-125b has been documented across the hair cycle, with functional assays indicating direct targeting of *FGF5* and *TNF-α*, two established regulators of hair length and growth kinetics [145]. Microarray comparisons between goat and sheep skin identified nine goat-enriched miRNAs, including mmu-miR-720 and mmu-miR-199b, suggesting lineage-specific regulatory programs [146]. Multi-omics integration expanded the cashmere goat miRNA repertoire by identifying 307 novel mature miRNAs and 72 differentially expressed candidates between growth and regression phases, providing a systems-level framework for follicular regulation [109]. Collectively, these data indicate dynamic miRNA remodeling during cyclic follicle development; however, the strength of evidence varies from correlative expression profiling to reporter-based target validation and in vitro functional perturbation, whereas conditional in vivo genetic models remain largely unavailable in goats.

Beyond hair cycling, miRNAs are increasingly implicated in coat color determination. Deep sequencing of white and black follicle samples identified miR-10b and miR-211 as color-associated candidates based on differential expression [147]. Comprehensive profiling detected 316 miRNAs linked to follicular development and secondary fiber growth, including 22 novel miRNAs (e.g., miR-368 and miR-668) potentially associated with pigmentation traits [148]. Functional stratification reveals heterogeneous levels of validation. For example, miR-200a exhibits differential expression between coat colors; gain- and loss-of-function assays in vitro demonstrated inverse regulation of *WNT5A* and *FZD4*, and antagomiR-200a administration in mice altered *WNT5A*/*FZD4* expression and melanin content in vivo [149]. Similarly, dual-luciferase assays confirmed direct targeting of *WNT3A* and *KITLG* by miR-27a, supported by coordinated mRNA and protein changes detected by qRT-PCR and Western blotting [150]. In Inner Mongolian cashmere goats, miR-129-5p expression inversely correlated with *TYR* abundance across breeds, suggesting repression of melanogenesis based primarily on expression and target prediction analyses [151]. By contrast, luciferase assays indicated regulatory interactions between miR-193b and *WNT10A*/*GNAI2*, although the reported positive association underscores the need for mechanistic clarification [152].

Taken together, current evidence supports a multilayered role for miRNAs in coordinating follicle cycling and pigmentation. Nevertheless, most studies rely on differential expression analyses, reporter assays, and in vitro perturbation experiments; rigorous in vivo genetic validation is still limited. A clear distinction among correlative profiling, direct target validation, and functional causality will be essential for consolidating the regulatory framework of miRNA-mediated control of goat hair follicle biology (Table 1).

## 7. miRNA Regulation of Hair Follicle Development in Sheep: From Wool Traits to Follicle Morphology

### 7.1. miRNA–mRNA Regulatory Networks in Sheep Hair Follicle Development

Ovine follicle morphogenesis and cycling are governed by multilayered miRNA–mRNA interactions. Functional resolution varies substantially across studies. For example, dual-luciferase assays demonstrated that miR-21 directly represses *CNKSR2*, *KLF3*, and *TNPO1*, while inverse seasonal expression patterns between miR-21 and its targets were observed in Merino and Small-tailed Han sheep, supporting a correlative role in cycling [153]. Target validation further identified *DLX3* as a miR-188 substrate in sheep fibroblasts and human keratinocytes; notably, the SNP c.1038_1039 insC modulates miR-188-mediated repression, linking post-transcriptional control to wool phenotypic variation [154].

Developmental profiling across embryonic (E65-E135) and postnatal stages identified 87 known and 446 novel differentially expressed miRNAs, with miR-23b and miR-133 targeting *WNT10A* and *ACVR1B*, respectively, based on prediction and reporter assays [155]. Similarly, miR-125b binding to *FGFR2* and *MXD4* was validated via 3′UTR assays, implicating it in fine wool and cashmere follicle growth [156]. Beyond linear regulation, ceRNA circuitry has emerged: XLOC005698 functions as a sponge for miR-3955-5p during secondary follicle morphogenesis [157], while the MSTRG.223165-miR-21-*SOX6* axis was supported by bioinformatic prediction and luciferase validation in Aohan fine-wool sheep [158]. CircRNA profiling further identified circ0003042 as a potential miR-432 sponge during fetal development, based primarily on computational prediction and differential expression [159]. Collectively, most conclusions are derived from integrated transcriptomics combined with reporter assays; in vivo genetic validation remains absent.

### 7.2. Signaling Pathway Modulation by miRNAs in Ovine Folliculogenesis

miRNAs intersect with canonical pathways governing epithelial–mesenchymal signaling. Six differentially expressed miRNAs (including oar-miR-103-3p and oar-miR-31-5p) were predicted to regulate Wnt pathway components, although evidence is largely bioinformatic [160]. High-throughput sequencing in China Merino sheep identified embryonic day 85 as critical for secondary follicle formation, implicating PRL and platelet activation pathways and highlighting candidate miRNAs (e.g., oar-miR-133, miR-376c-3p, miR-196b) based on expression profiling and pathway enrichment analyses [161]. Thus, pathway-level inferences currently rely predominantly on correlative datasets.

### 7.3. Cell-Level Functions of miRNAs in Sheep Hair Follicle Cells

At the cellular level, mechanistic depth varies. In ovine stem-like cells, miR-27a directly targets *PIK3R3*, *AKT*, and *MTOR*, suppressing phosphorylation and proliferation while inducing apoptosis, supported by molecular assays and functional perturbation [162]. In DPCs, miR-143 overexpression reduces *CUX1* and *KRT71* expression, linking luciferase-validated targeting to proliferation control and wool curvature [163]. miR-148/miR-10a targeting of *BMP7* was confirmed by reporter assays and Western blot; mimic transfection inhibited DPC proliferation in vitro [164]. These studies combine target validation with gain-of-function assays yet lack conditional in vivo models.

### 7.4. miRNAs and Wool Traits: Curvature, Density, and Fiber Quality

High-throughput sequencing and array-based profiling have identified numerous miRNAs associated with ovine wool traits; however, the depth of mechanistic validation varies substantially. During the wool cycle, Solexa sequencing combined with qRT-PCR demonstrated that miR-148b is enriched (>5-fold) in anagen relative to telogen in Tibetan sheep, supporting a correlative association with growth phase transition [99]. More broadly, spatiotemporal miRNA dynamics across developmental stages indicate regulatory involvement, yet most evidence derives from differential expression analyses rather than functional interrogation.

Wool curvature—a key determinant of lambskin quality—has been examined through integrative transcriptomics. Joint miRNA–mRNA profiling across large-, medium-, and small-wave follicles in lake lamb identified differentially expressed miRNAs potentially linked to waveform specification, based primarily on co-expression and target prediction [165]. Subsequent circRNA-miRNA network analysis in Hu sheep revealed 37 differentially expressed miRNAs and 114 circRNAs; in silico prediction (miRanda) suggested 129 circRNA-miRNA interactions, highlighting candidate regulatory pairs such as miR-125b-*CD34*, miR-181a-*FGF12/LMO3*, and miR-200b-*ZNF536* that may indirectly influence follicle architecture [166,167]. These conclusions rely largely on computational inference and correlative transcriptomics.

Illumina HiSeq profiling across distinct wave patterns in Hu sheep further identified candidate miRNAs (e.g., miR-143, miR-10a, let-7i, and several novel loci) associated with lambskin phenotype, again based on differential expression without in vivo functional validation [168]. In Tan sheep, comparative small RNA sequencing between lamb and adult skin uncovered 232 skin-enriched and 49 differentially expressed miRNAs; pathway enrichment implicated MAPK, WNT, and AMPK signaling, and a miR-432-*KRT83* negative regulatory module was proposed from target prediction and expression correlation analyses [169].

Collectively, current studies delineate extensive miRNA signatures linked to curvature, density, and fiber quality. Nevertheless, most inferences are grounded in expression profiling, bioinformatic target prediction, and network construction. Direct target validation (e.g., luciferase assays), in vitro gain- or loss-of-function experiments, and especially conditional in vivo genetic models remain limited. A clearer stratification of correlative versus causative evidence will be essential to establish mechanistic links between specific miRNAs and economically relevant wool phenotypes (Table 2).

## 8. Conserved Regulatory Networks, Species-Specific Adaptation, and Translational Perspectives of miRNAs in Hair Follicle Biology

Comparative analyses across mouse, human, goat, and sheep studies indicate that miRNA-mediated regulation of hair follicle biology is organized around a limited number of evolutionarily conserved regulatory modules, rather than isolated, pathway-specific effects. Multiple miRNAs repeatedly converge on key signaling nodes within the Wnt/β-catenin, BMP/TGF-β, MAPK, and FGF pathways, forming an interconnected regulatory architecture that underlies epithelial–mesenchymal communication, stem cell activation, and cyclic regeneration. For instance, miR-214 has been consistently shown to repress β-catenin signaling in mouse, human, and goat systems through direct targeting or epigenetic modulation, thereby influencing hair follicle stem cell dynamics and morphogenesis [79,98,141]. Similarly, the miR-29a/b1 cluster coordinately modulates both BMP and Wnt signaling by targeting BMPR1A and Wnt-related components, suggesting a role in synchronizing lineage progression rather than regulating a single pathway [81]. miR-31 and miR-31-5p further exemplify this integrative property by targeting genes associated with Wnt, BMP, and MAPK pathways, thereby contributing to the balance between proliferation and differentiation in both mouse and goat follicles [90,129]. In addition, conserved regulators of hair cycle transitions, such as miR-125b, modulate key growth-related genes, including FGF5 and TNF-α, across species, supporting a shared role in controlling follicular growth kinetics [145,156,166]. Collectively, these findings support a model in which miRNAs function as network-level regulators that coordinate crosstalk among signaling pathways, rather than acting as linear upstream or downstream effectors.

**Figure 6:**
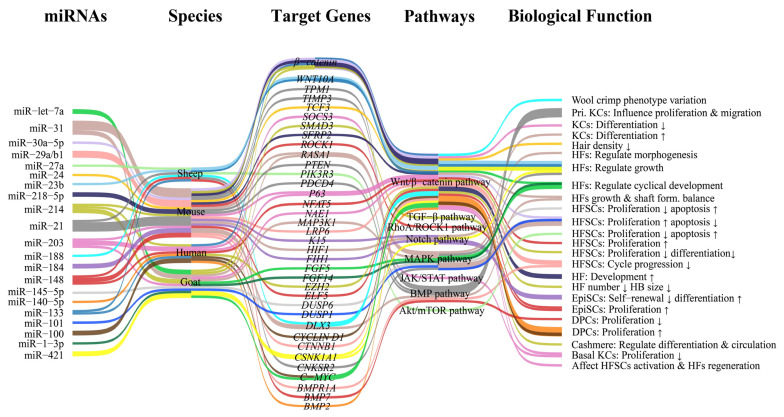
Systems-level model of miRNA-mediated regulation of hair follicle development: conserved network modules and species-specific rewiring. This figure presents a systems-level architecture of miRNA-mediated regulation in hair follicle development, integrating multilayered regulatory relationships among miRNAs, target genes, signaling pathways, and biological functions across species (mouse, human, goat, and sheep). Representative miRNAs are shown on the left, with corresponding target genes, involved signaling pathways (e.g., Wnt/β-catenin, BMP/TGF-β), and their functions in hair follicle biology indicated on the right. An upward arrow indicates increased biofunction, and a downward arrow indicates decreased biofunction.

Despite this conserved regulatory framework, substantial divergence emerges at the level of network configuration and phenotypic output across species (Figure 6). In mouse and human systems, miRNA function is primarily associated with fundamental processes such as stem cell maintenance, proliferation, and follicle morphogenesis, whereas in fiber-producing livestock, these regulatory networks are further adapted to control economically important traits. In cashmere goats, the regulatory landscape is characterized by enhanced complexity associated with secondary hair follicles, where multiple miRNAs, including let-7a, miR-199a-5p, miR-1-3p, and miR-130b-3p, exhibit stage-specific expression and converge on Wnt, FGF, and TGF-β signaling components to regulate follicle cycling and differentiation [103,105,106,138]. Regulatory interactions targeting WNT10A, FGFR2, and TGFβR2 highlight a pronounced emphasis on epithelial–mesenchymal signaling in secondary follicle morphogenesis [138,139]. In contrast, ovine studies suggest a shift toward regulation of fiber structural traits, where miRNAs such as miR-143, miR-125b, miR-181a, and miR-200b are associated with dermal papilla activity, keratin gene expression, and wool curvature, reflecting species-specific adaptation to fiber phenotype determination [163,166,167]. Additional evidence indicates that certain miRNAs, such as miR-432, may regulate structural proteins (e.g., KRT83) linked to wool morphology, further supporting divergence at the level of downstream targets [169]. These observations suggest that while upstream signaling pathways are largely conserved, miRNA regulatory networks undergo species-specific rewiring, resulting in distinct biological outputs that cannot be directly extrapolated from rodent models to livestock systems (Figure 6).

An additional layer of regulatory complexity is introduced by ceRNA networks, which have been widely reported in goat and sheep studies but exhibit substantial variability in experimental support. A subset of ceRNA interactions, such as the H19–miR-214–β-catenin axis and the lncRNA-599547–miR-15b–WNT10B pathway, are supported by multiple lines of evidence, including dual-luciferase assays, molecular interaction studies, and functional analyses, indicating a role in dermal papilla cell proliferation and inductive capacity [141,142]. Similarly, circRNA-mediated regulation of miRNAs (e.g., circRNA-1967–miR-93–LEF1) has been linked to lineage differentiation in secondary follicles [136]. However, a large proportion of reported ceRNA networks remain based on high-throughput sequencing and bioinformatic prediction with limited functional validation [109,110,118]. Given that effective ceRNA regulation requires precise stoichiometric relationships and sufficient expression levels, the physiological relevance of many predicted networks remains uncertain. Therefore, current evidence supports a stratified interpretation in which only a minority of ceRNA networks represent functionally validated regulatory axes, whereas the majority should be considered provisional.

From a translational perspective, the combination of conserved regulatory modules and species-specific adaptations provides both opportunities and constraints for practical application. Conserved miRNA–pathway interactions, particularly those involving Wnt/β-catenin signaling, represent promising targets for hair regeneration and treatment of follicular disorders. For example, miR-205, miR-218-5p, and miR-100 have been shown to promote hair regeneration through modulation of β-catenin signaling or related pathways, including via extracellular vesicle-mediated delivery [86,87,100]. In parallel, livestock studies have identified miRNAs associated with economically relevant traits, including fiber fineness (e.g., miR-2330), pigmentation (miR-200a and miR-27a), and follicle development (miR-21–SOX6 axis), suggesting potential applications in molecular breeding [111,149,150,158]. However, translation into practical strategies remains limited by several key challenges. Most notably, functional validation in vivo is scarce in large animal models, and the majority of mechanistic insights are derived from in vitro systems or correlative analyses. In addition, the biological significance of many ceRNA networks remains unclear, and the development of efficient, tissue-specific delivery systems for miRNA modulation is still in early stages. Finally, the inherent network complexity of miRNA regulation, in which a single miRNA may target multiple genes across pathways, introduces potential pleiotropic effects that complicate precise intervention. Addressing these limitations will require integration of genome editing, single-cell transcriptomics, and systems-level modeling to establish causal relationships and predict network behavior under physiological conditions.

Together, current evidence supports a hierarchical model in which conserved miRNA modules regulate core signaling pathways governing hair follicle development, while species-specific network rewiring shapes phenotypic diversity in fiber-producing animals (Figure 6). This framework provides a basis for integrating mechanistic understanding with translational applications but also underscores the need for more rigorous in vivo validation and systems-level analysis.

## 9. Conclusions

Hair follicle development represents a highly dynamic model of cyclical organogenesis, driven by tightly coordinated epithelial–mesenchymal interactions and spatiotemporally regulated signaling gradients. Accumulating evidence demonstrates that miRNAs function as essential post-transcriptional regulators within this system, acting not as primary determinants but as modulators that refine canonical signaling pathways. Both global disruption of miRNA biogenesis and functional studies of individual miRNAs consistently highlight their indispensable roles in follicular morphogenesis, stem cell regulation, and lineage specification. Key miRNAs, including miR-214, miR-31, miR-125b, and miR-203, modulate the intensity and timing of Wnt/β-catenin and related signaling pathways, thereby influencing progenitor cell fate decisions and structural gene expression. Importantly, these miRNAs operate as network-level buffering elements, stabilizing gene expression programs and ensuring robustness of hair follicle development and regeneration against intrinsic variability and environmental fluctuations.

During hair follicle cycling, miRNAs integrate extracellular cues with intracellular transcriptional programs to regulate stem cell quiescence, activation thresholds, and regenerative transitions, supporting a multilayered regulatory framework that extends beyond transcriptional control. However, significant challenges remain. Most mechanistic insights are derived from murine models, whereas studies in livestock species are largely limited to expression profiling, with insufficient in vivo functional validation. In addition, the combinatorial and network-level interactions among miRNAs—and their integration with other epigenetic regulators such as DNA methylation, histone modifications, and long non-coding RNAs—remain poorly defined. Future research should therefore prioritize systems-level approaches, cross-species functional validation, and integrative multi-omics analyses to establish causal regulatory networks and to fully realize the translational potential of miRNA-mediated modulation in both regenerative medicine and animal breeding.

## Figures and Tables

**Table 1:** miRNA functions closely related to the development of goat skin hair follicles.

miRNA	Evidence Type	Verification Type	Target Gene	Function	References
miR-let-7a	qRT-PCR, Western blot, luciferase reporter assays	In vitro	*C-myc*; *FGF5*	Regulates the cyclical development of hair follicles	[103]
chi-miR-199a-5p	qRT-PCR, Western blot, luciferase reporter assays	In vitro	*TGFβ2*	Potential delayed hair follicle development	[105]
miR-1-3p	qRT-PCR, Western blot, luciferase reporter assays	In vitro	*FGF14*	Regulates the cyclical development of hair follicles	[106]
miR-203	qRT-PCR, Western blot, luciferase reporter assays	In vitro	*DDOST*; *NAE1*	Regulates the hair follicles development	[107]
miR-873	qRT-PCR, high-throughput sequencing	In vitro	lnc108635596-*TGFβ1*/*BDNF*	Regulates the anagen/catagen transition of secondary follicles	[109]
miR-221-5p	qRT-PCR, high-throughput sequencing	In vitro	lnc000679-*WNT3*	Regulates cashmere development and circulation	[110]
miR-34a	qRT-PCR, high-throughput sequencing	In vitro	lnc000181-*GATA3*	Regulates cashmere development and circulation	[110]
miR-214-3p	qRT-PCR, high-throughput sequencing	In vitro	lnc000344-*SMAD3*	Regulates cashmere development and circulation	[110]
miR-2330	qRT-PCR, high-throughput sequencing	In vitro	lncRNA MSTRG14109.1-*KRT35*	Adjusts the fineness of cashmere	[111]
miR-2330	qRT-PCR, high-throughput sequencing	In vitro	circRNA452-*JUNB*	Adjusts the fineness of cashmere	[111]
miR-515-5p; miR-519e-5p	qRT-PCR, high-throughput sequencing	In vitro	lncRNA475083-*WNT3*; *NOTCH2*; *FGFR2*	Regulates cyclic activation and reconstruction of secondary follicles	[114]
chi-miR-1	qRT-PCR, high-throughput sequencing	In vitro	lncRNA MSTRG.1705.1	Regulates the growth and development of hair follicles	[115]
chi-miR-17-5p	qRT-PCR, high-throughput sequencing	In vitro	circRNA6854	Adjusts cashmere fineness	[119]
chi-miR-331-5p	qRT-PCR, high-throughput sequencing	In vitro	circRNA128	Adjusts cashmere fineness	[119]
chi-miR-27b; chi-miR-16b-3p	qRT-PCR, luciferase reporter assays	In vitro	circRNA3236	Regulates the growth and development of hair follicles	[120]
miR-27b-3p	qRT-PCR, luciferase reporter assays	In vitro	circRNA5712-*DLL4*	Regulates the growth and development of secondary follicles	[121]
chi-miR-421	qRT-PCR, high-throughput sequencing	In vitro	*CSNK1A1*	Regulates hair growth through the Wnt/Notch pathway	[124]
chi-miR-30e-5p	Luciferase reporter assays	In vitro	*DLL4*	Potentially regulates hair follicle development	[125]
chi-miR-30b-5p	Luciferase reporter assays	In vitro	*CaMKIIδ*	Inhibits DPC proliferation	[127]
miR-31-5p	qRT-PCR, Western blot, luciferase reporter assays	In vitro	*RASA1*; *MAP3K1*	Improves HFSC proliferation and inhibits apoptosis	[129]
miR-101	qRT-PCR, Western blot, luciferase reporter assays, flow cytometry	In vitro	*DUSP1*	Promotes HFSC proliferation and inhibits apoptosis	[130]
miR-145-5p	qRT-PCR, Western blot, luciferase reporter assays, flow cytometry	In vitro	*DUSP6*	Inhibits HFSCs and enhances apoptotic	[131]
miR-149-5p	qRT-PCR, Western blot, luciferase reporter assays, flow cytometry	In vitro	*CMTM3*	Promotes HFSC proliferation and inhibits apoptosis	[132]
miR-1	qRT-PCR, Western blot, luciferase reporter assays, flow cytometry	In vitro	*IGF1R*; *LEF1*	Enhances HFSC differentiation	[134]
miR-22-5p	qRT-PCR, Western blot, luciferase reporter assays, flow cytometry	In vitro	*LEF1*	Inhibits HFSC proliferation	[135]
miR-93-3p	qRT-PCR, Western blot, luciferase reporter assays, flow cytometry, RNA pull-down assay	In vitro	circRNA-1967-*LEF1*	Promotes HFSC differentiation	[136]
miR-153-3p	qRT-PCR, luciferase reporter assays, flow cytometry	In vitro	circRNA-0100-*KLF5*	Promotes HFSC differentiation	[137]
chi-miR-370-3p	qRT-PCR, Western blot, luciferase reporter assays	In vitro	*TGFβR2*; *FGFR2*	Inhibits the proliferation of epithelial cells and fibroblasts	[139]
chi-miR-144-5p	High-throughput sequencing	In vitro	XR310320.3-*HOXC8*	Activation of HFSCs	[140]
novel-624	High-throughput sequencing	In vitro	XR311077.2-*RSPO1*	Activation of HFSCs	[140]
chi-miR-214-3p	High-throughput sequencing, qRT-PCR, Western blot, luciferase reporter assays	In vitro	lncRNA-H19-*β-Catenin*	Promotes DPC proliferation	[141]
miR-15b-5p	qRT-PCR, Western blot, luciferase reporter assays, RNA pull-down assay	In vitro	lncRNA-599547-*WNT10B*	Contributes to DPCs’ inductive property	[142]
miR-195	High-throughput sequencing	In vitro	*CHP1*; *SMAD2*; *FZD6*; *SIAH1*	Regulates the initiation process of hair follicles	[144]
miR-200a	qRT-PCR, Western blot, luciferase reporter assays, immunohistochemistry staining, transgenic models	In vivo and in vitro	*WNT5A*; *FZD4*	Regulates the pigmentation of the fur color	[149]
miR-27a	qRT-PCR, Western blot, luciferase reporter assays	In vitro	*WNT3A*; *KITLG*	Adjusts the cashmere wool color	[150]
miR-129-5p	qRT-PCR, Western blot, luciferase reporter assays	In vitro	*TYR*	Affects the formation of coat color	[151]

**Table 2:** miRNA functions closely related to the development of sheepskin hair follicles.

miRNA	Evidence Type	Verification Type	Target Gene	Function	References
miR-21	qRT-PCR, luciferase reporter assays	In vitro	*CNKSR2*; *KLF3*; *TNPO1*	Participates in the development of hair follicles	[153]
miR-188	qRT-PCR, luciferase reporter assays	In vitro	*DLX3*	Wool crimp phenotype variation	[154]
miR-23b	qRT-PCR, Western blot, luciferase reporter assays	In vitro	*WNT10A*	Regulates the development of hair follicles	[155]
miR-125b	qRT-PCR, Western blot, luciferase reporter assays	In vitro	*FGFR2*; *MXD4*	Regulates the growth and development of hair follicles	[156]
miR-3955-5p	High-throughput sequencing	In vitro	XLOC005698	Regulation of secondary follicles morphogenesis	[156]
miR-27a	qRT-PCR, Western blot, luciferase reporter assays	In vitro	*PIK3R3*; *AKT*; *MTOR*	Inhibits HFSC proliferation and promotes apoptosis	[162]
miR-143	qRT-PCR, Western blot, luciferase reporter assays, flow cytometry	In vitro	*CUX1*;	Inhibits DPC proliferation	[163]
miR-143	qRT-PCR, Western blot, luciferase reporter assays, flow cytometry	In vitro	*KRT71*	Regulates HFs growth	[163]
miR-148; miR-10a	qRT-PCR, Western blot, luciferase reporter assays	In vitro	*BMP7*	Inhibits DPC proliferation	[164]
miR-125b	qRT-PCR, High-throughput sequencing	In vitro	*CD34*	Indirectly affects hair follicle development and wool curvature	[166]
miR-181a	qRT-PCR, High-throughput sequencing	In vitro	*FGF12*; *LMO3*	Indirectly affects hair follicle development and wool curvature	[166]
miR-200b	qRT-PCR, High-throughput sequencing	In vitro	*ZNF536*	Indirectly affects hair follicle development and wool curvature	[166]
miR-432	qRT-PCR, Western blot, luciferase reporter assays	In vitro	*KRT83*	Closely related to the formation of curly wool	[169]

## Data Availability

No new data were created or analyzed in this study.
